# Supplementary material for: Analysis of PPARγ Signaling Activity in Psoriasis
Source: Int J Mol Sci. 2021 Aug 10;22(16):8603. doi: 10.3390/ijms22168603 (PMC8395241; doi:10.3390/ijms22168603)
Supplement: Supplementary file 1 [file ijms-22-08603-s001.zip › Supplemental materials_Analysis of PPARg signaling activity in psoriasis/Pathway models/Models images and html files/Differentiation of psoriatic T cells/10991.html]

STAT1


# Protein STAT1

|  |  |
| --- | --- |
| URN | urn:agi-llid:6772 |
| Total Entities | 0 |
| Connectivity | 5078 |
| Name | STAT1 |
| Description | signal transducer and activator of transcription 1, 91kDa |
| Notes | The protein encoded by this gene is a member of the STAT protein family. In response to cytokines and growth factors, STAT family members are phosphorylated by the receptor associated kinases, and then form homo- or heterodimers that translocate to the cell nucleus where they act as transcription activators. This protein can be activated by various ligands including interferon-alpha, interferon-gamma, EGF, PDGF and IL6. This protein mediates the expression of a variety of genes, which is thought to be important for cell viability in response to different cell stimuli and pathogens. Two alternatively spliced transcript variants encoding distinct isoforms have been described. [provided by RefSeq, Jul 2008] |
| Primary Cell Localization | Nucleus |
| Class | Transcription factor |
| ObjectType | Protein |

---

|  |  |
| --- | --- |
| Pathway | Thyroid Hormones Common non-Genomic Effects in Hyperthyroidism |
|  | PRL and CSH Action in Mammary Gland |
|  | Thrombopoietin Receptors Signaling in Platelet Maturation |
|  | Natural Killer Cell Activation |
|  | CD8+ T-Cell Activation |
|  | T-Cell Cytotoxic Mediated Cell Death |
|  | Th1-Cell Differentiation |
|  | AHR Signaling in Treg and Dendritic Cells Function |
|  | AHR Signaling in Tr1 Cells Function |
|  | CXC Chemokine Receptor Signaling |
|  | CC Chemokine Receptor Signaling |
|  | Plasmin Effects in Inflammation |
|  | Basophil Activation |
|  | Eosinophil Survival by Cytokine Signaling |
|  | Mast-Cell Activation via IgE Signaling |
|  | Mast-Cell Activation without Degranulation through CXCR4 Signaling |
|  | Mast-Cell Activation without Degranulation |
|  | Mast-Cells De Novo Synthesized Mediators via IgE Independent Signaling |
|  | Macrophage M1 Lineage |
|  | CD16/CD14 Proinflammatory Monocyte Activation |
|  | AHR Signaling in M1 Macrophages Function |
|  | AXL Receptor Inhibits Macrophages and Dendritic Cells Function |
|  | Neutrophil Recruitment and Priming |
|  | Vascular Endothelial Cell Activation by Cytokines |
|  | Vascular Endothelial Cell Activation by Blood Coagulation Factors |
|  | Vascular Endothelial Cell Activation by Growth Factors |
|  | Nociception Expression Targets Signaling |
|  | CCR1 Expression Targets |
|  | IL6 Expression Targets -> Nociception |
|  | G0/G1 Phase Transition |
|  | Proteins Involved in Atherosclerosis |
|  | Th1-Cells Activation and Proliferation in Atherosclerosis |
|  | Cardiomyocyte Hypertrophy |
|  | Hypertrophic Cardiomyopathy |
|  | Endothelial Cell Dysfunction in Pulmonary Hypertension |
|  | Proteins Involved in Myocardial Ischemia |
|  | Preconditioning Ischemia |
|  | Lymphocyte Mediated Myocardial Injury in Myocarditis |
|  | SOCS in Negative Feedback Regulation in Myocarditis |
|  | Hair Follicle Keratinocyte Apoptosis |
|  | Atopic Dermatitis |
|  | Melanoma |
|  | Apoptosis Block in Melanoma Cell |
|  | Proteins Involved in Melanoma |
|  | T-Cells Differentiation Block in Psoriasis |
|  | IFN-gamma/TNF-alpha Mediated Cell Proliferation in Psoriasis |
|  | Dendritic Cell Activation in Systemic Lupus Erythematosis |
|  | Proteins Involved in Systemic Lupus Erythematosus |
|  | Th1-Cell Function in Systemic Lupus Erythematosus |
|  | B-Cell Activation in Systemic Lupus Erythematosus |
|  | Vitiligo |
|  | T-Cell Cytotoxic Response against Melanocytes in Vitiligo |
|  | Plasmacytoid Dendritic Cell in Diabetes Mellitus Type 1 |
|  | Toll-like Receptors in beta-Cell |
|  | Tubular Cell Dysfunction in Progressive Diabetic Nephropathy |
|  | Proteins Involved in Diabetic Nephropathy |
|  | beta-Cell Destruction through Cytokines in Diabetes Mellitus |
|  | beta-Cell Destruction in Diabetes Mellitus Type 1 |
|  | Nitric Oxide Effects on beta-Cell |
|  | Hashimoto's Thyroiditis |
|  | Proteins Involved in Hypothyroidism |
|  | Proteins Involved in Celiac Disease |
|  | CD4+T-Cell Response in Celiac Disease |
|  | Metastatic Colorectal Cancer |
|  | Th1-Cell Activation in Crohn's Disease |
|  | OipA Signaling in Helicobacter Pylori Infection |
|  | Proteins Involved in Ulcerative Colitis |
|  | B-Cell Acute Lymphoblastic Leukemia |
|  | Diffuse Large-B-Cell Lymphoma |
|  | Primary Mediastinal B-cell Lymphoma (PMBL) Subtype |
|  | Follicular Lymphoma |
|  | Proteins Involved in Hodgkin Lymphoma |
|  | IL6/IGF1/VEGFA Signaling in Multiple Myeloma |
|  | Multiple Myeloma |
|  | T-Cell Acute Lymphoblastic Leukemia |
|  | Eosinophil Survival in Asthma |
|  | Mast-Cell Activation in Asthma |
|  | Osteoclast Activation in Gout |
|  | Neutrophil Recruitment in Sinovium in Gout |
|  | Dendritic Cell Dysfunction in Psoriatic Arthritis |
|  | Osteoclast Activation in Psoriatic Arthritis |
|  | Keratinocyte Activation in Psoriatic Arthritis |
|  | Proteins Involved in Psoriatic Arthritis |
|  | Osteoclast Activation in Rheumatoid Arthritis |
|  | Skin Fibrosis |
|  | CCR5 Signaling in Macrophages |
|  | CD4+ T-Cell Death in HIV |
|  | Diabetes Induced Periodontitis |
|  | Glioblastoma, Primary |
|  | Glioblastoma, Secondary |
|  | Astrocytoma |
|  | IL6/IL12 Signaling Activates Immune System in Multiple Sclerosis |
|  | Proteins Involved in Breast Cancer Related to ESR1 Signaling Pathway |
|  | Proteins Involved in Breast Cancer Related to ERBB2/VEGFR/Akt Signaling Pathway |
|  | Proteins Involved in Breast Cancer Related to IGF1R/Akt Signaling Pathway |
|  | Cholesteatoma |
|  | Prostate Cancer |
|  | RNase L Signaling in Prostate Cancer |
|  | Proteins Involved in Prostate Cancer |
|  | CDKN2B-AS1 in Epigenetic Regulation of Glaucoma |
|  | Aminoglycosides and Cisplatin Induced Ototoxicity (Mouse Model) |
|  | Proteins Involved in Hearing Loss |
|  | Cytokines Trigger Otitis Media |
|  | Proteins Involved in Chronic Obstructive Pulmonary Disease |
|  | Polycystins Mutation Effects in Cystic Kidney Disease |
|  | Endothelial Cell Dysfunction in Pyelonephritis |
|  | AREG -> STAT Expression Targets |
|  | Noreadrenaline/Gq Expression Targets |
|  | Epinephrine/Gq Expression Targets |
|  | Aryl Hydrocarbon Receptor Genomic and non-Genomic Signaling |
|  | BTC -> STAT Expression Targets |
|  | IFNG -> ARRB1/STAT1 Signaling |
|  | CD2 Expression Targets |
|  | CD22 -> STAT Expression Targets |
|  | CD8 -> NFATC Expression Targets |
|  | CD80 -> ATF/CREB/CREBBP Expression Targets |
|  | CD86 -> ATF/CREB/CREBBP Expression Targets |
|  | CXCL12 Expression Targets |
|  | CCL3 Expression Targets |
|  | CCL16 Expression Targets |
|  | CCL5 Expression Targets |
|  | CCL7 Expression Target |
|  | CCL15 Expression Targets |
|  | CCL2 Expression Targets |
|  | IL8 Expression Targets |
|  | CSF3 Expression Targets |
|  | CSF2 -> STAT Expression Targets |
|  | Clozapine Induced Granulocytopenia |
|  | EGF -> STAT Expression Targets |
|  | EGFR/ERBB -> STAT Signaling |
|  | EREG -> STAT Expression Targets |
|  | Erythropoietin -> STAT Expression Targets |
|  | F2 -> STAT1/NF-kB Expression Targets |
|  | PLG -> STAT1/NF-kB Expression Targets |
|  | FGF2 -> STAT Expression Targets |
|  | FGF10 -> STAT Expression Targets |
|  | FGF18 -> STAT Expression Targets |
|  | FGF1 -> STAT Expression Targets |
|  | FGFR1 -> STAT Signaling |
|  | FGFR3 -> STAT Signaling |
|  | GH1/GHR -> STAT Expression Targets |
|  | CSH1/GHR Expression Targets |
|  | CSH1/PRLR Expression Targets |
|  | GH1/PRLR Expression Targets |
|  | HGF -> STAT Expression Targets |
|  | HGFR -> STAT Signaling |
|  | Receptors and Adaptor Proteins Activated in Cancer |
|  | Proteins with Altered Expression in Cancer-Associated Sustaining of Proliferative Signaling |
|  | G0/G1 Cell Cycle Phase Transition Activation in Cancer |
|  | N2 Neutrophils in Tumor-Promoting Inflammation and Tumor Progression |
|  | VEGF Independent Angiogenesis in Cancer |
|  | Myeloid Derived Suppressor Cells in Cancer Immune Escape |
|  | IDO1 in Cancer Immune Escape |
|  | IGF2 -> STAT Expression Targets |
|  | IGF1 -> STAT Expression Targets |
|  | Insulin -> STAT Expression Targets |
|  | CD2 -> STAT Signaling |
|  | InsulinR -> STAT Signaling |
|  | IGF1R -> STAT Signaling |
|  | IFNLR1-> STAT/NFKB Signaling |
|  | IFNW1/IFNR Expression Target |
|  | IFNA1/IFNR Expression Targets |
|  | IFNB1/IFNR Expression Targets |
|  | IFNG/IFNR Expression Targets |
|  | IL31R -> STAT Signaling |
|  | IL15R -> STAT Signaling |
|  | IL10R -> STAT Signaling |
|  | IL13R -> STAT Signaling |
|  | IL3R -> STAT Signaling |
|  | IL27R -> STAT Signaling |
|  | IL7R -> STAT Signaling |
|  | IL9R -> STAT Signaling |
|  | IL5R -> STAT Signaling |
|  | IL6R -> STAT Signaling |
|  | IL2R -> STAT Signaling |
|  | IL12R -> STAT Signaling |
|  | IL21R -> STAT Signaling |
|  | IL7 Expression Targets |
|  | IL13 Expression Targets |
|  | IL21 Expression Targets |
|  | IL15 Expression Targets |
|  | IL12B Expression Targets |
|  | IL3 Expression Targets |
|  | IL6 Expression Targets |
|  | IL10 Expression Targets |
|  | IL5 Expression Targets |
|  | IL9 Expression Targets |
|  | IL31 Expression Targets |
|  | IL2 Expression Targets |
|  | UrokinaseR -> STAT Signaling |
|  | Leptin -> STAT Expression Targets |
|  | EGFR-> Expression Targets in Lymphoid System and Blood |
|  | NRG1 -> STAT Expression Targets |
|  | PTGER4 -> Expression Targets in Nerve Tissue |
|  | PDGF -> STAT Expression Targets |
|  | PDGFR -> STAT Signaling |
|  | CSF2 -> STAT Signaling |
|  | KIT -> STAT Signaling |
|  | GAST Expression Targets in GAST Expression Targets |
|  | CCL11 Expression Targets |
|  | AVP/Gq -> STAT Expression Targets |
|  | AVP/Gs -> STAT Expression Targets |
|  | AGT -> STAT Expression Targets |
|  | PRL/PRLR Expression Targets |
|  | PRL/GHR -> STAT Expression Targets |
|  | ProlactinR -> STAT Signaling |
|  | PDCD1 -> STAT Expression Targets |
|  | ALK -> STAT Signaling |
|  | EphrinR -> STAT Signaling |
|  | CSF1 -> STAT Expression Targets |
|  | MDK/PTN Expression Targets |
|  | KITLG -> STAT Expression Targets |
|  | EphrinR Expression Targets |
|  | Ras-GAP Regulation Signaling |
|  | AXL Receptor Tyrosine Kinase Signaling |
|  | EGFR Signaling |
|  | TCR -> STAT Expression Targets |
|  | T-Cell Receptor -> STAT Signaling |
|  | TGFA -> STAT Expression Targets |
|  | LTA Expression Targets |
|  | TNF -> STAT Expression Targets |
|  | TNFRSF1A -> STAT Signaling |
|  | Thrombopoietin -> STAT Expression Targets |
|  | PLAU -> STAT1 Expression Targets |
|  | VEGFA -> STAT Expression Targets |
|  | VEGFR -> STAT Signaling |
|  | PPAR Psoriasis |
|  | pprarg neg, uknown targets, ps-positive |
|  | prarg neg,ukn expres targets, ps-positive |
|  | PPARG negative targets |
|  | pparg expr target\_regulators |
|  | Neighbors of disease exacerbation |
|  | Neighbors of tumor progression |
|  | Neighbors of cancer progression |
|  | Neighbors of inflammatory status |
|  | Neighbors of overall survival |
|  | Neighbors of disease severity |
|  | Neighbors of tumor size |
|  | Neighbors of lesion size |
|  | Neighbors of mortality |
|  | Neighbors of infarct size |
|  | Neighbors of functional recovery |
|  | Neighbors of bacterial load |
|  | Neighbors of cancer survival |
|  | Neighbors of graft survival |
|  | Neighbors of lethality |
|  | Neighbors of tumor regression |
|  | Neighbors of disease-free survival |
|  | Neighbors of tumor response |
|  | Neighbors of macrophage count |
|  | Neighbors of bone mass |
|  | Neighbors of tumor recurrence |
|  | Neighbors of tumor promotion |
|  | Neighbors of morbidity |
|  | Neighbors of fibrosis progression |
|  | Neighbors of bone mineral density |
|  | Neighbors of radioresistance |
|  | Neighbors of lesion area |
|  | Neighbors of cell density |
|  | Neighbors of early mortality |
|  | Neighbors of viral load |
|  | Neighbors of leukocyte count |
|  | Neighbors of cancer risk |
|  | Neighbors of tumor aggressiveness |
|  | Neighbors of tumor resistance |
|  | Neighbors of osteoclast number |
|  | Neighbors of cancer recurrence |
|  | Neighbors of T-cell count |
|  | Neighbors of radiosensitivity |
|  | Neighbors of recurrence rate |
|  | Neighbors of disease exacerbation |
|  | Neighbors of tumor progression |
|  | Neighbors of cancer progression |
|  | Neighbors of inflammatory status |
|  | Neighbors of overall survival |
|  | Neighbors of disease severity |
|  | Neighbors of tumor size |
|  | Neighbors of lesion size |
|  | Neighbors of mortality |
|  | Neighbors of infarct size |
|  | Neighbors of functional recovery |
|  | Neighbors of bacterial load |
|  | Neighbors of cancer survival |
|  | Neighbors of graft survival |
|  | Neighbors of lethality |
|  | Neighbors of tumor regression |
|  | Neighbors of disease-free survival |
|  | Neighbors of tumor response |
|  | Neighbors of macrophage count |
|  | Neighbors of bone mass |
|  | Neighbors of tumor recurrence |
|  | Neighbors of tumor promotion |
|  | Neighbors of morbidity |
|  | Neighbors of fibrosis progression |
|  | Neighbors of bone mineral density |
|  | Neighbors of radioresistance |
|  | Neighbors of lesion area |
|  | Neighbors of cell density |
|  | Neighbors of early mortality |
|  | Neighbors of viral load |
|  | Neighbors of leukocyte count |
|  | Neighbors of cancer risk |
|  | Neighbors of tumor aggressiveness |
|  | Neighbors of tumor resistance |
|  | Neighbors of osteoclast number |
|  | Neighbors of cancer recurrence |
|  | Neighbors of T-cell count |
|  | Neighbors of radiosensitivity |
|  | Neighbors of recurrence rate |
|  | Neighbors of cell development |
|  | Neighbors of immune response |
|  | Neighbors of cell population |
|  | Neighbors of T-cell development |
|  | Neighbors of inflammatory response |
|  | Neighbors of wound healing |
|  | Neighbors of angiogenesis |
|  | Neighbors of innate immune response |
|  | Neighbors of cell function |
|  | Neighbors of macrophage activation |
|  | Neighbors of cell formation |
|  | Neighbors of adaptive immune response |
|  | Neighbors of osteoclast development |
|  | Neighbors of epithelial to mesenchymal transition |
|  | Neighbors of immunity |
|  | Neighbors of cell proliferative response |
|  | Neighbors of chemotaxis |
|  | Neighbors of dendritic cell differentiation |
|  | Neighbors of cell survival |
|  | Neighbors of osteoclast differentiation |
|  | Neighbors of cell phenotype |
|  | Neighbors of T-cell proliferation |
|  | Neighbors of tissue remodeling |
|  | Neighbors of cell adhesion |
|  | Neighbors of T-cell activation |
|  | Neighbors of endothelial cell proliferation |
|  | Neighbors of tumor growth |
|  | Neighbors of dendritic cell development |
|  | Neighbors of tissue repair |
|  | Neighbors of cell count |
|  | Neighbors of cell infiltration |
|  | Neighbors of cell interaction |
|  | Neighbors of SMC proliferation |
|  | Neighbors of lumen formation |
|  | Neighbors of endothelial cell migration |
|  | Neighbors of adipogenesis |
|  | Neighbors of lipid storage |
|  | Neighbors of cell damage |
|  | Neighbors of pregnancy |
|  | Neighbors of monocyte differentiation |
|  | Neighbors of aging |
|  | Neighbors of cell motility |
|  | Neighbors of bone resorption |
|  | Neighbors of monocyte migration |
|  | Neighbors of fibroblast proliferation |
|  | Neighbors of adipocyte differentiation |
|  | Neighbors of macrophage migration |
|  | Neighbors of microglial activation |
|  | Neighbors of cell migration |
|  | Neighbors of cancer growth |
|  | Neighbors of stem cell proliferation |
|  | Neighbors of smooth muscle cell migration |
|  | Neighbors of hepatic regeneration |
|  | Neighbors of transendothelial migration |
|  | Neighbors of phagocytosis |
|  | Neighbors of immune system activation |
|  | Neighbors of leukocyte migration |
|  | Neighbors of osteoclast formation |
|  | Neighbors of cell invasion |
|  | Neighbors of hemopoiesis |
|  | Neighbors of bone remodeling |
|  | Neighbors of ossification |
|  | Neighbors of T-cell function |
|  | Neighbors of immune system function |
|  | Neighbors of autophagy |
|  | Neighbors of monocyte adhesion |
|  | Neighbors of transcription activation |
|  | Neighbors of senescence |
|  | Neighbors of T-cell response |
|  | Neighbors of tumor immunity |
|  | Neighbors of hepatocyte proliferation |
|  | Neighbors of cell transdifferentiation |
|  | Neighbors of neuronal death |
|  | Neighbors of cell growth |
|  | Neighbors of ROS generation |
|  | Neighbors of epithelial cell proliferation |
|  | Neighbors of cancer cell growth |
|  | Neighbors of sensitization |
|  | Neighbors of hemato-encephalic barrier |
|  | Neighbors of macrophage function |
|  | Neighbors of cellular immune response |
|  | Neighbors of cellular senescence |
|  | Neighbors of colony formation |
|  | Neighbors of macrophage differentiation |
|  | Neighbors of cell differentiation |
|  | Neighbors of cell homeostasis |
|  | Neighbors of psoriasis |
|  | Neighbors of inflammatory disease |
|  | Neighbors of rheumatoid arthritis |
|  | Neighbors of atherosclerosis |
|  | Neighbors of fibrosis |
|  | Neighbors of colitis |
|  | Neighbors of arthritis |
|  | Neighbors of inflammatory bowel disease |
|  | Neighbors of injury |
|  | Neighbors of metastasis |
|  | Neighbors of carcinogenesis |
|  | Neighbors of multiple sclerosis |
|  | Neighbors of chronic inflammation |
|  | Neighbors of inflammation |
|  | Neighbors of tumor microenvironment |
|  | Neighbors of obesity |
|  | Neighbors of diabetes mellitus |
|  | Neighbors of asthma |
|  | Neighbors of pneumonia |
|  | Neighbors of liver fibrosis |
|  | Neighbors of chronic obstructive pulmonary disease |
|  | Neighbors of myocardial infarction |
|  | Neighbors of leukocyte infiltration |
|  | Neighbors of reperfusion injury |
|  | Neighbors of airway inflammation |
|  | Neighbors of hepatitis |
|  | Neighbors of insulin resistance |
|  | Neighbors of infection |
|  | Neighbors of vascular injury |
|  | Neighbors of renal injury |
|  | Neighbors of macrophage infiltration |
|  | Neighbors of liver injury |
|  | Neighbors of neuroinflammation |
|  | Neighbors of sepsis |
|  | Neighbors of death |
|  | Neighbors of type 1 diabetes |
|  | Neighbors of encephalomyelitis |
|  | Neighbors of autoimmune disease |
|  | Neighbors of ischemic stroke |
|  | Neighbors of dermatitis |
|  | Neighbors of neutrophil infiltration |
|  | Neighbors of diabetic nephropathy |
|  | Neighbors of lung injury |
|  | Neighbors of immunopathology |
|  | Neighbors of systemic sclerosis |
|  | Neighbors of hepatocellular carcinoma |
|  | Neighbors of autoimmunity |
|  | Neighbors of acute lung injury |
|  | Neighbors of gastric cancer |
|  | Neighbors of experimental autoimmune encephalomyelitis |
|  | Neighbors of colorectal cancer |
|  | Neighbors of cardiac remodeling |
|  | Neighbors of breast cancer |
|  | Neighbors of ischemia |
|  | Neighbors of systemic lupus erythematosus |
|  | Neighbors of melanoma |
|  | Neighbors of collagen-induced arthritis |
|  | Neighbors of interstitial fibrosis |
|  | Neighbors of kidney disease |
|  | Neighbors of heart failure |
|  | Neighbors of lung disease |
|  | Neighbors of pulmonary fibrosis |
|  | Neighbors of cardiovascular disease |
|  | Neighbors of stroke |
|  | Neighbors of cancer |
|  | Neighbors of neoplasm |
|  | Neighbors of neointima |
|  | Neighbors of vasculitis |
|  | Neighbors of atrophy |
|  | Neighbors of respiratory hypersensitivity |
|  | Neighbors of myocardial fibrosis |
|  | Neighbors of synovitis |
|  | Neighbors of coronary artery disease |
|  | Neighbors of liver disease |
|  | Neighbors of prostate cancer |
|  | Neighbors of ovarian cancer |
|  | Neighbors of pancreatic cancer |
|  | Neighbors of hypertension |
|  | Neighbors of allograft rejection |
|  | Neighbors of macrophage |
|  | Neighbors of fibroblast |
|  | Neighbors of hepatocyte |
|  | Neighbors of endothelial cell |
|  | Neighbors of immunocompetent cell |
|  | Neighbors of myeloid cell |
|  | Neighbors of insulin-secreting cell |
|  | Neighbors of T-cell |
|  | Neighbors of lung |
|  | Neighbors of colorectal region |
|  | Neighbors of NF-kB family |
|  | Neighbors of Jun/Fos |
|  | Neighbors of NFKBI |
|  | Neighbors of STAT family |
|  | Neighbors of IFNAR ligand |
|  | Neighbors of cytokine |
|  | Neighbors of interferon |
|  | Neighbors of JAK |
|  | Neighbors of Ras GTPase |
|  | Neighbors of ovalbumin |
|  | Neighbors of LXR |
|  | Neighbors of lectin |
|  | Neighbors of proteasome endopeptidase complex |
|  | Neighbors of STAT5 |
|  | Neighbors of retinoid-X receptor subfamily |
|  | Neighbors of ITG |
|  | Neighbors of TORC1 |
|  | Neighbors of RA receptor |
|  | Neighbors of histone acetyltransferase |
|  | Neighbors of deacetylase |
|  | comon ps\_pos, pprarg\_neg targets |
|  | Model of PPARG signaling in psoriasis |
|  | PPARG negative regulators and targets |
|  | Model of PPARG related pathways in psoriasis (short version) |
|  | New Pathway (3) |
|  | sars-cov no binding |
|  | IFN signaling in alveolar cells |
|  | Dendritic Cell Activation in COVID19 |
|  | Figure 3\_Mixed network of dexamethasone targets |
|  | Figure 3\_Mixed network of dexamethasone targets |
|  | 1\_Differentiation of psoriatic T cells |
|  | 1\_Formation of cysts in polycystic kidney disease |
|  | 1\_Endothelial cells stimulate vasoconstriction and thrombosis in pulmonary hypertension |
|  | 2\_Cardiomyocyte hypertrophy |
|  | 2\_2\_Eosinophilia and neutrophilia in asthma: eosinophil apoptosis |
|  | 4\_Inflammatory mediators inhibit GATA signaling in erythropoiesis |
|  | 1\_Antigen-presenting cells promote islet dysfunction and immune system activation in T1D |
|  | 3\_Islet beta-cell destruction in T1D |
|  | drug-target pairs direct regulation |
|  | drug-target moltransport |
|  | Differentiation of psoriatic T cells |

---

|  |  |
| --- | --- |
| Group | Genes Associated with Systemic Lupus Erythematosus |
|  | other partners for SARS |

---

|  |  |
| --- | --- |
| Source | curated |
|  | ResNet |
|  | GO component |

---

|  |  |
| --- | --- |
| MedScan ID | 6772 |

---

|  |  |
| --- | --- |
| LocusLink ID | 6772 |
|  | 20846 |
|  | 25124 |
|  | 98183 |
|  | 510814 |
|  | 282084 |
|  | 30768 |

---

|  |  |
| --- | --- |
| Alias | CANDF7 |
|  | Stat1 |
|  | Stat I |
|  | STAT91 |
|  | Signal transducer and activator of transcription I |
|  | 91 kDa signal transducer and activator of transcription I |
|  | STAT1 transcription factor |
|  | signal transducer and activator of transcription 1, 91kDa |
|  | transcription factor components p91/p84 |
|  | 2010005J02Rik |
|  | signal transducer and activator of transcription 1, 91kD |
|  | Signal transducer and activator of transcription 1-alpha/beta |
|  | 91 kDa signal transducer and activator of transcription 1 |
|  | DD6G4-4 |
|  | transcription factor STAT1 |
|  | AA408197 |
|  | OTTHUMP00000165046 |
|  | PIR:A46159 |
|  | OTTHUMP00000205845 |
|  | signal transducer and activator of transcription 1 |
|  | Transcription factor ISGF-3 components p91/p84 |
|  | DKFZp686b04100 |
|  | OTTHUMP00000165047 |
|  | IMD31A |
|  | IMD31B |
|  | IMD31C |
|  | ISGF-3 |
|  | hornerin |
|  | signal transducer and activator of transcription-1 |
|  | STAT91s |
|  | interferon-dependent positive-acting transcription factor ISGF-3 |
|  | RIKEN cDNA 1110033K19 gene |
|  | transcription factor ISGF III |
|  | transcription factor ISGF-3 |
|  | AV011854 |
|  | expressed sequence AV011854 |
|  | 1110033K19Rik |

---

|  |  |
| --- | --- |
| GO ID | 0031730 |
|  | 0001228 |
|  | 0003700 |
|  | 0000981 |
|  | 0000979 |
|  | 0000978 |
|  | 0045296 |
|  | 0003690 |
|  | 0019899 |
|  | 0035035 |
|  | 0042393 |
|  | 0035257 |
|  | 1990841 |
|  | 0042803 |
|  | 0051721 |
|  | 0070491 |
|  | 0005164 |
|  | 0044389 |
|  | 0007259 |
|  | 0008015 |
|  | 0032869 |
|  | 0035458 |
|  | 0071346 |
|  | 0071407 |
|  | 0019221 |
|  | 0006952 |
|  | 0051607 |
|  | 0043542 |
|  | 0060333 |
|  | 0038114 |
|  | 0070106 |
|  | 0070757 |
|  | 0070102 |
|  | 0038113 |
|  | 0010742 |
|  | 0072162 |
|  | 0072136 |
|  | 0046725 |
|  | 0043124 |
|  | 0016525 |
|  | 0001937 |
|  | 0003340 |
|  | 0072308 |
|  | 0000122 |
|  | 0002230 |
|  | 0045648 |
|  | 0032727 |
|  | 0002053 |
|  | 0051770 |
|  | 0048661 |
|  | 0045944 |
|  | 0007221 |
|  | 0045893 |
|  | 0042981 |
|  | 0042127 |
|  | 0060334 |
|  | 0061326 |
|  | 0051591 |
|  | 0034097 |
|  | 0042542 |
|  | 0035456 |
|  | 0009612 |
|  | 0007584 |
|  | 0043434 |
|  | 0033209 |
|  | 0060337 |
|  | 0016032 |
|  | 0030424 |
|  | 0005737 |
|  | 0005829 |
|  | 0030425 |
|  | 0000790 |
|  | 0005730 |
|  | 0005654 |
|  | 0005634 |
|  | 0048471 |
|  | 0032991 |
|  | 0003677 |
|  | 0043565 |
|  | 0006919 |
|  | 0071345 |
|  | 0071222 |
|  | 0031663 |
|  | 0034240 |
|  | 0008284 |
|  | 0006355 |
|  | 0009617 |
|  | 0042493 |
|  | 0043330 |
|  | 0032496 |
|  | 0034340 |
|  | 0007165 |
|  | 0006351 |
|  | 0006357 |
|  | 0014070 |
|  | 0000983 |
|  | 0000982 |
|  | 0001047 |
|  | 0005509 |
|  | 0005515 |
|  | 0004871 |
|  | 0019048 |
|  | 0006915 |
|  | 0006917 |
|  | 0060397 |
|  | 0006366 |
|  | 0009615 |
|  | 0060338 |
|  | 0007249 |
|  | 0044419 |
|  | 0007260 |
|  | 0010553 |
|  | 0010628 |
|  | 0005062 |
|  | 0006350 |
|  | 0045449 |
|  | 0007262 |
|  | 0009613 |
|  | 0000074 |
|  | 0007242 |

---

|  |  |
| --- | --- |
| KEGG ID | hsa:6772 |
|  | rno:25124 |
|  | mmu:20846 |

---

|  |  |
| --- | --- |
| MedLine Reference | 11024034 |
|  | 12191995 |
|  | 12477932 |
|  | 12527914 |
|  | 12584205 |
|  | 12960068 |
|  | 1299057 |
|  | 14642652 |
|  | 14678947 |
|  | 14984365 |
|  | 15262323 |
|  | 15590660 |
|  | 15630703 |
|  | 15659221 |
|  | 15948243 |
|  | 15956343 |
|  | 16027122 |
|  | 16212889 |
|  | 16555329 |
|  | 16616937 |
|  | 9716657 |
|  | 10200816 |
|  | 10349636 |
|  | 10364154 |
|  | 10364426 |
|  | 10510345 |
|  | 10526573 |
|  | 10562320 |
|  | 10629051 |
|  | 10640742 |
|  | 10781830 |
|  | 10817963 |
|  | 10851046 |
|  | 10851049 |
|  | 10851050 |
|  | 10851062 |
|  | 10865997 |
|  | 10867663 |
|  | 10899912 |
|  | 10946258 |
|  | 11042159 |
|  | 11076861 |
|  | 11086033 |
|  | 11217851 |
|  | 11323675 |
|  | 11371356 |
|  | 11390995 |
|  | 11493533 |
|  | 11544199 |
|  | 11554754 |
|  | 11579100 |
|  | 11881154 |
|  | 11884587 |
|  | 11932417 |
|  | 11971185 |
|  | 11972023 |
|  | 12006410 |
|  | 12006974 |
|  | 12007403 |
|  | 12018856 |
|  | 12033784 |
|  | 12138178 |
|  | 12162464 |
|  | 12167251 |
|  | 12171910 |
|  | 12198138 |
|  | 12219085 |
|  | 12232043 |
|  | 12270932 |
|  | 12364588 |
|  | 12370359 |
|  | 12407025 |
|  | 12426001 |
|  | 12438372 |
|  | 12438448 |
|  | 12441126 |
|  | 12461178 |
|  | 12466851 |
|  | 12526039 |
|  | 12551969 |
|  | 12624267 |
|  | 12634107 |
|  | 12671680 |
|  | 12679810 |
|  | 12686553 |
|  | 12727921 |
|  | 12730885 |
|  | 12811839 |
|  | 12816762 |
|  | 12832402 |
|  | 12859963 |
|  | 12865406 |
|  | 12923053 |
|  | 12925694 |
|  | 12957148 |
|  | 14522949 |
|  | 14600148 |
|  | 14602726 |
|  | 14617019 |
|  | 14656722 |
|  | 14657353 |
|  | 14662340 |
|  | 14662881 |
|  | 14670297 |
|  | 14688355 |
|  | 14699080 |
|  | 14730360 |
|  | 14732359 |
|  | 14733929 |
|  | 14734769 |
|  | 14738323 |
|  | 14764699 |
|  | 14871928 |
|  | 14976252 |
|  | 14977926 |
|  | 14978477 |
|  | 15073186 |
|  | 15128825 |
|  | 15188379 |
|  | 15189833 |
|  | 15213094 |
|  | 15226272 |
|  | 15238607 |
|  | 15240659 |
|  | 15240691 |
|  | 15240699 |
|  | 15246962 |
|  | 15322115 |
|  | 15356124 |
|  | 15356135 |
|  | 15452265 |
|  | 15477229 |
|  | 15485925 |
|  | 15491990 |
|  | 15522878 |
|  | 15541348 |
|  | 15585884 |
|  | 15618518 |
|  | 15634877 |
|  | 15713743 |
|  | 15719366 |
|  | 15778340 |
|  | 15784679 |
|  | 15814715 |
|  | 15850655 |
|  | 15851485 |
|  | 15852015 |
|  | 15905519 |
|  | 15905569 |
|  | 15919828 |
|  | 15919906 |
|  | 15956575 |
|  | 15964802 |
|  | 15983048 |
|  | 16014513 |
|  | 16024814 |
|  | 16046307 |
|  | 16081786 |
|  | 16116224 |
|  | 16141072 |
|  | 16141073 |
|  | 16148142 |
|  | 16148148 |
|  | 16200070 |
|  | 16210330 |
|  | 16251407 |
|  | 16254350 |
|  | 16260600 |
|  | 16260741 |
|  | 16284791 |
|  | 16315087 |
|  | 16388483 |
|  | 16424049 |
|  | 16472772 |
|  | 16482512 |
|  | 16507120 |
|  | 16585592 |
|  | 16618773 |
|  | 16634641 |
|  | 16670015 |
|  | 16670771 |
|  | 16688530 |
|  | 16709859 |
|  | 16709865 |
|  | 16717119 |
|  | 16728283 |
|  | 16731906 |
|  | 16734559 |
|  | 16764725 |
|  | 16782051 |
|  | 16785492 |
|  | 16843267 |
|  | 16857984 |
|  | 16906167 |
|  | 17032168 |
|  | 17078933 |
|  | 17133483 |
|  | 7490523 |
|  | 7515712 |
|  | 7545930 |
|  | 7567467 |
|  | 7579387 |
|  | 8140422 |
|  | 8530075 |
|  | 8593242 |
|  | 8608597 |
|  | 8608598 |
|  | 8626752 |
|  | 8631837 |
|  | 8675207 |
|  | 8805627 |
|  | 8833906 |
|  | 9022056 |
|  | 9069288 |
|  | 9092506 |
|  | 9119818 |
|  | 9126479 |
|  | 9229117 |
|  | 9287210 |
|  | 9342212 |
|  | 9548487 |
|  | 9638363 |
|  | 9695817 |
|  | 9828143 |
|  | 9846495 |
|  | 9887329 |
|  | 10067896 |
|  | 10358045 |
|  | 10446176 |
|  | 10490649 |
|  | 10506573 |
|  | 10602027 |
|  | 10652206 |
|  | 10692450 |
|  | 10764778 |
|  | 10792030 |
|  | 10805787 |
|  | 10820245 |
|  | 10848577 |
|  | 10848598 |
|  | 10918587 |
|  | 10954736 |
|  | 10961983 |
|  | 10973496 |
|  | 10982844 |
|  | 11152457 |
|  | 11226159 |
|  | 11238845 |
|  | 11248027 |
|  | 11257227 |
|  | 11278462 |
|  | 11278865 |
|  | 11294897 |
|  | 11301323 |
|  | 11418623 |
|  | 11452125 |
|  | 11722592 |
|  | 11752148 |
|  | 11777927 |
|  | 11804954 |
|  | 11815625 |
|  | 11835405 |
|  | 11839738 |
|  | 11843291 |
|  | 11886859 |
|  | 11909852 |
|  | 11909970 |
|  | 11927559 |
|  | 11932384 |
|  | 11981818 |
|  | 12039028 |
|  | 12048190 |
|  | 12070153 |
|  | 12130529 |
|  | 12161037 |
|  | 12165521 |
|  | 12191570 |
|  | 12223098 |
|  | 12232802 |
|  | 12356736 |
|  | 12388709 |
|  | 12426389 |
|  | 12459177 |
|  | 12539042 |
|  | 12590259 |
|  | 12610144 |
|  | 12629155 |
|  | 12634403 |
|  | 12637327 |
|  | 12722480 |
|  | 12734330 |
|  | 12748293 |
|  | 12759242 |
|  | 12777975 |
|  | 12788789 |
|  | 12804771 |
|  | 12807916 |
|  | 12817007 |
|  | 12846741 |
|  | 12855573 |
|  | 12855578 |
|  | 12856330 |
|  | 12867595 |
|  | 12917439 |
|  | 12923054 |
|  | 12948935 |
|  | 12963127 |
|  | 12967644 |
|  | 14500749 |
|  | 14557668 |
|  | 14599788 |
|  | 14623896 |
|  | 14654078 |
|  | 14654702 |
|  | 14674010 |
|  | 14688135 |
|  | 14690454 |
|  | 14702039 |
|  | 14746803 |
|  | 14751560 |
|  | 14755057 |
|  | 14963018 |
|  | 1496401 |
|  | 14976049 |
|  | 15010467 |
|  | 1502203 |
|  | 15055356 |
|  | 15123634 |
|  | 15165826 |
|  | 15210729 |
|  | 15217838 |
|  | 15221897 |
|  | 15280488 |
|  | 15284024 |
|  | 15467722 |
|  | 15494521 |
|  | 15604419 |
|  | 15650219 |
|  | 15703780 |
|  | 15778351 |
|  | 15780933 |
|  | 15795279 |
|  | 15824515 |
|  | 15825084 |
|  | 15919942 |
|  | 15979846 |
|  | 15996661 |
|  | 15998520 |
|  | 16148108 |
|  | 16189514 |
|  | 16195225 |
|  | 16223733 |
|  | 16236267 |
|  | 16254375 |
|  | 16257975 |
|  | 16260419 |
|  | 16318585 |
|  | 16388135 |
|  | 16467883 |
|  | 16481475 |
|  | 16501077 |
|  | 16511123 |
|  | 16512786 |
|  | 16571725 |
|  | 16572323 |
|  | 16574066 |
|  | 16585190 |
|  | 16628196 |
|  | 16773129 |
|  | 16799645 |
|  | 16897667 |
|  | 16918696 |
|  | 16934001 |
|  | 16935931 |
|  | 16940534 |
|  | 16951379 |
|  | 17030574 |
|  | 17075845 |
|  | 17145596 |
|  | 17182865 |
|  | 7514165 |
|  | 7543024 |
|  | 7657660 |
|  | 7690989 |
|  | 7885841 |
|  | 8232552 |
|  | 8549654 |
|  | 8621447 |
|  | 8631962 |
|  | 8943351 |
|  | 8986769 |
|  | 9135145 |
|  | 9205132 |
|  | 9284918 |
|  | 9344858 |
|  | 9355737 |
|  | 9373149 |
|  | 9373245 |
|  | 9452495 |
|  | 9484840 |
|  | 9584171 |
|  | 9630226 |
|  | 9724754 |
|  | 9804857 |
|  | 9843502 |
|  | 9880553 |
|  | 9925928 |
|  | 9989503 |
|  | 12419823 |
|  | 10702714 |
|  | 11442634 |
|  | 15140960 |
|  | 15489334 |
|  | 16306601 |
|  | 16878149 |
|  | 16904741 |
|  | 16978698 |
|  | 17227821 |
|  | 17237409 |
|  | 17266440 |
|  | 17287281 |
|  | 17346710 |
|  | 17371985 |
|  | 17386941 |
|  | 17442890 |
|  | 17486072 |
|  | 17490620 |
|  | 17523873 |
|  | 17538964 |
|  | 17804871 |
|  | 8125298 |
|  | 10754318 |
|  | 11034357 |
|  | 11310846 |
|  | 12223448 |
|  | 12444163 |
|  | 12872135 |
|  | 15972639 |
|  | 16301649 |
|  | 16563502 |
|  | 16689660 |
|  | 17065231 |
|  | 17121994 |
|  | 17200147 |
|  | 17227866 |
|  | 17229692 |
|  | 17234735 |
|  | 17237438 |
|  | 17293456 |
|  | 17307940 |
|  | 17424888 |
|  | 17473223 |
|  | 17475277 |
|  | 17507688 |
|  | 17513775 |
|  | 17533179 |
|  | 17549733 |
|  | 17634555 |
|  | 17689680 |
|  | 10188224 |
|  | 15350851 |
|  | 15454281 |
|  | 15749807 |
|  | 16269269 |
|  | 17116388 |
|  | 17251186 |
|  | 17293493 |
|  | 7519422 |
|  | 9144559 |

---

|  |  |
| --- | --- |
| Organism | Homo sapiens {Organism urn:agi-taxid:9606} |
|  | Mus musculus {Organism urn:agi-taxid:10090} |
|  | Rattus norvegicus {Organism urn:agi-taxid:10116} |
|  | Homo sapiens |
|  | Mus musculus |
|  | Rattus norvegicus |
|  | Bos taurus |
|  | Danio rerio |

---

|  |  |
| --- | --- |
| Mouse chromosome position | 1 26.81 cM |
|  | 1 25.9 cM |

---

|  |  |
| --- | --- |
| OMIM ID | 600555 |
|  | 614892 |
|  | 614162 |
|  | 613796 |
|  | 209950 |

---

|  |  |
| --- | --- |
| Rat chromosome position | 9q22 |
|  | 9q31-q32 |

---

|  |  |
| --- | --- |
| Hugo ID | 11362 |
|  | HGNC:11362 |

---

|  |  |
| --- | --- |
| Human chromosome position | 2q32.2 |

---

|  |  |
| --- | --- |
| Swiss-Prot Accession | P42224 |
|  | P42224.2 |
|  | A0A087WSP5 |
|  | Q8C8M3 |
|  | Q8CFQ1 |
|  | Q8C3V4 |
|  | Q99K94 |
|  | Q8C497 |
|  | P42225.1 |
|  | Q6P6Q7 |
|  | F1M9D6 |
|  | Q9QXK0 |
|  | A8K989 |
|  | B2RCA0 |
|  | D2KFR8 |
|  | D3DPI7 |
|  | Q53S88 |
|  | Q53XW4 |
|  | Q68D00 |
|  | Q9UDL5 |
|  | J3KPM9 |
|  | P42225 |
|  | Q67C41 |
|  | Q3TW11 |
|  | Q3V3D6 |
|  | Q91Y52 |
|  | Q9D323 |
|  | O70406 |
|  | Q7TP57 |
|  | STAT1\_HUMAN |
|  | B2RCA0\_HUMAN |
|  | Q53XW4\_HUMAN |
|  | Q68D00\_HUMAN |
|  | Q6P6Q7\_RAT |
|  | Q8C3V4\_MOUSE |
|  | Q99K94\_MOUSE |

---

|  |  |
| --- | --- |
| PIR ID | A46159 |

---

|  |  |
| --- | --- |
| GenBank ID | NC\_000002 |
|  | XR\_001738915 |
|  | XR\_001738914 |
|  | XM\_017004783 |
|  | XP\_016860272 |
|  | NM\_007315 |
|  | NP\_009330 |
|  | XM\_006712718 |
|  | XP\_006712781 |
|  | NM\_139266 |
|  | NP\_644671 |
|  | NG\_008294 |
|  | AC067945 |
|  | AAY24183 |
|  | AH008323 |
|  | AAF04559 |
|  | AX354554 |
|  | CAD22974 |
|  | AY366464 |
|  | AAR13284 |
|  | AY865620 |
|  | AAW56072 |
|  | CH471058 |
|  | EAX10850 |
|  | EAX10851 |
|  | EAX10852 |
|  | EAX10853 |
|  | EAX10854 |
|  | EAX10855 |
|  | CS542449 |
|  | CAM91174 |
|  | CS542451 |
|  | CAM91175 |
|  | HQ284032 |
|  | U18662 |
|  | U18663 |
|  | U18664 |
|  | U18665 |
|  | U18666 |
|  | U18667 |
|  | U18668 |
|  | U18669 |
|  | U18670 |
|  | AI992149 |
|  | AK096686 |
|  | AK225853 |
|  | AK292604 |
|  | BAF85293 |
|  | AK315002 |
|  | BAG37497 |
|  | BC002704 |
|  | AAH02704 |
|  | BC141921 |
|  | AAI41922 |
|  | BG699050 |
|  | BT007241 |
|  | AAP35905 |
|  | BU738296 |
|  | BX400908 |
|  | CN410740 |
|  | CR749636 |
|  | CAH18430 |
|  | CR998778 |
|  | DA313984 |
|  | EB385742 |
|  | GU211347 |
|  | ADA59516 |
|  | GU211348 |
|  | ADA59517 |
|  | M97935 |
|  | AAB64012 |
|  | M97936 |
|  | P42224 |
|  | NC\_000067 |
|  | NM\_009283 |
|  | NP\_033309 |
|  | NM\_001205314 |
|  | NP\_001192243 |
|  | XM\_006495811 |
|  | XP\_006495874 |
|  | NM\_001205313 |
|  | NP\_001192242 |
|  | XM\_017319464 |
|  | XP\_017174953 |
|  | XM\_017319463 |
|  | XP\_017174952 |
|  | NM\_001357627 |
|  | AC123752 |
|  | AF349678 |
|  | AAK48844 |
|  | CH466548 |
|  | EDK99964 |
|  | EDK99965 |
|  | EDK99966 |
|  | EDK99967 |
|  | EDK99968 |
|  | U80267 |
|  | AK008127 |
|  | AK018544 |
|  | BAB31265 |
|  | AK039458 |
|  | BAC30355 |
|  | AK041599 |
|  | BAE43313 |
|  | AK041814 |
|  | BAE43314 |
|  | AK046517 |
|  | BAC32766 |
|  | AK082706 |
|  | BAC38579 |
|  | AK084855 |
|  | BAC39293 |
|  | AK088874 |
|  | AK157263 |
|  | BAE34017 |
|  | AK157458 |
|  | AK159887 |
|  | BAE35456 |
|  | BC004808 |
|  | AAH04808 |
|  | BC042551 |
|  | AAH42551 |
|  | BC057690 |
|  | BX513160 |
|  | U06924 |
|  | AAA19454 |
|  | P42225 |
|  | NC\_005108 |
|  | NM\_032612 |
|  | NP\_116001 |
|  | NM\_001034164 |
|  | NP\_001029336 |
|  | AC\_000077 |
|  | AABR07067607 |
|  | AABR07073481 |
|  | AAHX01059848 |
|  | AAHX01059849 |
|  | AC094675 |
|  | CH473965 |
|  | EDL99092 |
|  | AA901120 |
|  | AF034252 |
|  | AF053767 |
|  | AAC08438 |
|  | AF205604 |
|  | AAF20200 |
|  | BC062079 |
|  | AAH62079 |
|  | BM386875 |
|  | CF109416 |
|  | CV075250 |
|  | DY564252 |
|  | FQ227362 |
|  | FQ231334 |
|  | FQ232551 |
|  | ABBA01062392 |
|  | XM\_006495812 |
|  | XM\_006495813 |
|  | XM\_005246815 |
|  | XM\_005246816 |
|  | EU832073 |
|  | AAHY01006307 |
|  | AAHY01006306 |
|  | XP\_005246872 |
|  | XP\_005246873 |
|  | AMYH02005442 |
|  | AC\_000023 |
|  | XP\_006495876 |
|  | XP\_006495875 |
|  | AC\_000134 |
|  | ACE86735 |
|  | NC\_018913 |
|  | AF182311 |
|  | ACE87421 |
|  | AABR06060248 |
|  | AABR06060249 |
|  | AABR06060250 |
|  | AF182314 |
|  | EU831980 |
|  | NT\_005403 |
|  | NW\_921585 |
|  | NW\_001838863 |
|  | AC\_000045 |
|  | CR601119 |
|  | CR618286 |
|  | Q67C41 |
|  | NT\_039170 |
|  | NW\_001030680 |
|  | Q3TW11 |
|  | Q3V3D6 |
|  | Q8C3V4 |
|  | Q8C497 |
|  | Q8C8M3 |
|  | Q8CFQ1 |
|  | Q91Y52 |
|  | Q99K94 |
|  | Q9D323 |
|  | NW\_047815 |
|  | NW\_001084882 |
|  | O70406 |
|  | Q6P6Q7 |
|  | Q7TP57 |
|  | Q9QXK0 |
|  | Q53XW4 |
|  | Q9UDL5 |
|  | Q68D00 |
|  | AAH57690 |
|  | Q6PF86 |
|  | AABR03068246 |
|  | Q53S88 |
|  | U92116 |
|  | AW214029 |
|  | AA960121 |
|  | AAC02821 |
|  | NW\_000166 |
|  | BM239586 |
|  | 2996209 |
|  | 38304005 |
|  | 6636498 |
|  | 81861373 |
|  | 81869420 |
|  | 81885263 |
|  | 1174458 |
|  | 123781007 |
|  | 123783857 |
|  | 12858298 |
|  | 13435936 |
|  | 13898647 |
|  | 26333275 |
|  | 26338161 |
|  | 26349879 |
|  | 26351313 |
|  | 27502700 |
|  | 34785478 |
|  | 458710 |
|  | 74178727 |
|  | 74183076 |
|  | 74186815 |
|  | 74204784 |
|  | 81871537 |
|  | 81876394 |
|  | 81876464 |
|  | 81878027 |
|  | 81880102 |
|  | 81881308 |
|  | 81892594 |
|  | 81899350 |
|  | AA408197 |
|  | 119631255 |
|  | 119631256 |
|  | 119631257 |
|  | 119631260 |
|  | 12803735 |
|  | 2281071 |
|  | 2507413 |
|  | 30583321 |
|  | 38194550 |
|  | 51476968 |
|  | 57790280 |
|  | 62988796 |
|  | 74708726 |
|  | 74724461 |
|  | 74725554 |
|  | 74726678 |
|  | 74727062 |
|  | NT\_086633 |
|  | 122131798 |
|  | 154426144 |
|  | 45385786 |
|  | AAI51379 |
|  | AAS59770 |
|  | AY488117 |
|  | BC151378 |
|  | NC\_007300 |
|  | NW\_001494645 |
|  | Q05FF2 |
|  | XM\_001253473 |
|  | XP\_001253474 |
|  | 3687402 |
|  | 38173885 |
|  | 82071168 |
|  | 82186677 |
|  | 94733815 |
|  | AAH60933 |
|  | AJ005692 |
|  | BC045863 |
|  | BC060933 |
|  | BX548072 |
|  | CAA06676 |
|  | CAK11455 |
|  | NC\_007133 |
|  | NM\_131480 |
|  | NP\_571555 |
|  | NW\_001512391 |
|  | O93598 |
|  | Q6P943 |
|  | 119631258 |
|  | 119631259 |
|  | 148667548 |
|  | 148667549 |
|  | 148667550 |
|  | 148667551 |
|  | 148667552 |
|  | 149046199 |

---

|  |  |
| --- | --- |
| Swiss-Prot ID | STAT1\_HUMAN |
|  | Q8CFQ1\_MOUSE |
|  | Q9QXK0\_RAT |
|  | Q6P6Q7\_RAT |
|  | Q8C3V4\_MOUSE |
|  | Q99K94\_MOUSE |
|  | B2RCA0\_HUMAN |
|  | Q53XW4\_HUMAN |
|  | Q68D00\_HUMAN |
|  | D3DPI7\_HUMAN |

---

|  |  |
| --- | --- |
| Cell Localization | Cytoplasm |
|  | Nucleus |

---

|  |  |
| --- | --- |
| Ensembl ID | ENSG00000115415 |
|  | ENSP00000354394.3 |
|  | ENST00000361099.7 |
|  | ENSP00000376136.3 |
|  | ENST00000392322.7 |
|  | ENST00000540176.5 |
|  | ENSMUSG00000026104 |
|  | ENSMUSP00000141132.1 |
|  | ENSMUST00000186057.6 |
|  | ENSMUSP00000140875.1 |
|  | ENSMUST00000186857.6 |
|  | ENSMUSP00000066743.7 |
|  | ENSMUST00000070968.13 |
|  | ENSMUSP00000140518.1 |
|  | ENSMUST00000186574.6 |
|  | ENSRNOG00000014079 |
|  | ENSRNOP00000019465.6 |
|  | ENSRNOT00000019465.7 |
|  | ENSRNOP00000019465 |
|  | ENSRNOT00000019465 |
|  | ENSP00000376136 |
|  | ENST00000392322 |
|  | ENSP00000376137 |
|  | ENSMUSP00000141132 |
|  | ENST00000392323 |
|  | ENSMUSP00000066743 |
|  | ENSMUSP00000140518 |
|  | ENSMUST00000070968 |
|  | ENSMUST00000186057 |
|  | ENSMUST00000186574 |
|  | ENSP00000354394 |
|  | ENSMUST00000186857 |
|  | ENST00000361099 |
|  | ENSMUSP00000140875 |

---

|  |  |
| --- | --- |
| MGI ID | MGI:103063 |
|  | 103063 |
|  | 2138078 |

---

|  |  |
| --- | --- |
| RGD ID | 3771 |

---

|  |  |
| --- | --- |
| Unigene ID | Hs.642990 |
|  | Mm.277406 |
|  | Rn.33229 |
|  | Hs.743244 |
|  | Mm.487336 |
|  | Hs.715518 |
|  | Hs.724418 |
|  | Hs.708051 |
|  | Hs.470943 |
|  | Hs.651258 |
|  | Bt.33292 |
|  | Dr.120105 |
|  | Hs.699271 |

---

|  |  |
| --- | --- |
| Homologene ID | 21428 |

---

|  |  |
| --- | --- |
| Shape | O-vertex |

---

|  |  |
| --- | --- |
| IPI ID | IPI00030781 |
|  | IPI00218188 |
|  | IPI00956843 |
|  | IPI00213245 |
|  | IPI00655247 |
|  | IPI00467004 |
|  | IPI00795482 |
|  | IPI00797149 |
|  | IPI00652052 |
|  | IPI00626843 |
|  | IPI00382229 |

---

|  |  |
| --- | --- |
| Microarray ID | 101465\_at |
|  | AFFX-HUMISGF3A/M97935\_MA\_at |
|  | AFFX-HUMISGF3A/M97935\_5\_at |
|  | 200887\_s\_at |
|  | AFFX-HUMISGF3A/M97935\_3\_at |

---

|  |  |
| --- | --- |
| Homo sapiens Chromosome position | 2q32.2 |

---

|  |  |
| --- | --- |
| Ensembl Transcript ID | ENST00000361099 |
|  | ENST00000409465 |
|  | ENST00000392323 |
|  | ENST00000392322 |

---

|  |  |
| --- | --- |
| ENSEMBL | ENSG00000115415 |

---

|  |  |
| --- | --- |
| FunctionalClass | hematopoietin/interferon-class (D200-domain) cytokine receptor signal transducer |
|  | protein binding |
|  | transcription factor |
|  | DNA binding |
|  | calcium ion binding |
|  | signal transducer |

---

|  |  |
| --- | --- |
| Bos taurus Chromosome position | 2 |

---

|  |  |
| --- | --- |
| Mus musculus Chromosome position | 1 25.9 cM |
|  | 1 |

---

|  |  |
| --- | --- |
| KEGG pathway | Jak-STAT signaling pathway |

---

|  |  |
| --- | --- |
| ProteinAtlas SubcellularLocation | Main location: Nucleus but not nucleoli;Cytoplasm; Other location: ; Expression type: APE; Reliability: High |

---

|  |  |
| --- | --- |
| Rattus norvegicus Chromosome position | 9q31-q32 |

---

|  |  |
| --- | --- |
| Ensembl Gene ID | ENSG00000115415 |
|  | LRG\_111 |

---

|  |  |
| --- | --- |
| ProteinAtlas NormalTissue | Tissue: gall bladder; Cell type: glandular cells; Level: Low; Expression type: APE; Reliability: Low |
|  | Tissue: lateral ventricle; Cell type: glial cells; Level: None; Expression type: APE; Reliability: Low |
|  | Tissue: lung; Cell type: pneumocytes; Level: None; Expression type: APE; Reliability: Low |
|  | Tissue: appendix; Cell type: lymphoid tissue; Level: Medium; Expression type: APE; Reliability: Low |
|  | Tissue: cerebellum; Cell type: cells in molecular layer; Level: None; Expression type: APE; Reliability: Low |
|  | Tissue: stomach |
|  | upper; Cell type: glandular cells; Level: Low; Expression type: APE; Reliability: Low |
|  | Tissue: tonsil; Cell type: squamous epithelial cells; Level: Medium; Expression type: APE; Reliability: Low |
|  | Tissue: vagina; Cell type: squamous epithelial cells; Level: None; Expression type: APE; Reliability: Low |
|  | Tissue: ovary; Cell type: ovarian stroma cells; Level: None; Expression type: APE; Reliability: Low |
|  | Tissue: placenta; Cell type: trophoblastic cells; Level: Low; Expression type: APE; Reliability: Low |
|  | Tissue: skin; Cell type: epidermal cells; Level: None; Expression type: APE; Reliability: Low |
|  | Tissue: kidney; Cell type: cells in tubules; Level: Medium; Expression type: APE; Reliability: Low |
|  | Tissue: adrenal gland; Cell type: glandular cells; Level: None; Expression type: APE; Reliability: Low |
|  | Tissue: cervix |
|  | uterine; Cell type: squamous epithelial cells; Level: Low; Expression type: APE; Reliability: Low |
|  | Tissue: spleen; Cell type: cells in white pulp; Level: Medium; Expression type: APE; Reliability: Low |
|  | Tissue: urinary bladder; Cell type: urothelial cells; Level: Medium; Expression type: APE; Reliability: Low |
|  | Tissue: skeletal muscle; Cell type: myocytes; Level: None; Expression type: APE; Reliability: Low |
|  | Tissue: appendix; Cell type: glandular cells; Level: Low; Expression type: APE; Reliability: Low |
|  | Tissue: bone marrow; Cell type: hematopoietic cells; Level: Medium; Expression type: APE; Reliability: Low |
|  | Tissue: breast; Cell type: glandular cells; Level: Low; Expression type: APE; Reliability: Low |
|  | Tissue: bronchus; Cell type: respiratory epithelial cells; Level: Low; Expression type: APE; Reliability: Low |
|  | Tissue: cerebellum; Cell type: cells in granular layer; Level: None; Expression type: APE; Reliability: Low |
|  | Tissue: cerebellum; Cell type: Purkinje cells; Level: None; Expression type: APE; Reliability: Low |
|  | Tissue: cerebral cortex; Cell type: glial cells; Level: None; Expression type: APE; Reliability: Low |
|  | Tissue: cerebral cortex; Cell type: neuronal cells; Level: None; Expression type: APE; Reliability: Low |
|  | Tissue: cervix, uterine; Cell type: glandular cells; Level: None; Expression type: APE; Reliability: Low |
|  | Tissue: cervix, uterine; Cell type: squamous epithelial cells; Level: Low; Expression type: APE; Reliability: Low |
|  | Tissue: colon; Cell type: glandular cells; Level: Low; Expression type: APE; Reliability: Low |
|  | Tissue: duodenum; Cell type: glandular cells; Level: Low; Expression type: APE; Reliability: Low |
|  | Tissue: epididymis; Cell type: glandular cells; Level: Low; Expression type: APE; Reliability: Low |
|  | Tissue: esophagus; Cell type: squamous epithelial cells; Level: Low; Expression type: APE; Reliability: Low |
|  | Tissue: fallopian tube; Cell type: glandular cells; Level: None; Expression type: APE; Reliability: Low |
|  | Tissue: heart muscle; Cell type: myocytes; Level: None; Expression type: APE; Reliability: Low |
|  | Tissue: hippocampus; Cell type: glial cells; Level: None; Expression type: APE; Reliability: Low |
|  | Tissue: hippocampus; Cell type: neuronal cells; Level: None; Expression type: APE; Reliability: Low |
|  | Tissue: kidney; Cell type: cells in glomeruli; Level: Low; Expression type: APE; Reliability: Low |
|  | Tissue: lateral ventricle; Cell type: neuronal cells; Level: None; Expression type: APE; Reliability: Low |
|  | Tissue: liver; Cell type: bile duct cells; Level: None; Expression type: APE; Reliability: Low |
|  | Tissue: liver; Cell type: hepatocytes; Level: None; Expression type: APE; Reliability: Low |
|  | Tissue: lung; Cell type: macrophages; Level: Medium; Expression type: APE; Reliability: Low |
|  | Tissue: lymph node; Cell type: germinal center cells; Level: Medium; Expression type: APE; Reliability: Low |
|  | Tissue: lymph node; Cell type: non-germinal center cells; Level: Medium; Expression type: APE; Reliability: Low |
|  | Tissue: nasopharynx; Cell type: respiratory epithelial cells; Level: Low; Expression type: APE; Reliability: Low |
|  | Tissue: oral mucosa; Cell type: squamous epithelial cells; Level: Medium; Expression type: APE; Reliability: Low |
|  | Tissue: ovary; Cell type: follicle cells; Level: None; Expression type: APE; Reliability: Low |
|  | Tissue: pancreas; Cell type: exocrine glandular cells; Level: Low; Expression type: APE; Reliability: Low |
|  | Tissue: pancreas; Cell type: islets of Langerhans; Level: Low; Expression type: APE; Reliability: Low |
|  | Tissue: parathyroid gland; Cell type: glandular cells; Level: None; Expression type: APE; Reliability: Low |
|  | Tissue: placenta; Cell type: decidual cells; Level: Low; Expression type: APE; Reliability: Low |
|  | Tissue: prostate; Cell type: glandular cells; Level: Medium; Expression type: APE; Reliability: Low |
|  | Tissue: rectum; Cell type: glandular cells; Level: Low; Expression type: APE; Reliability: Low |
|  | Tissue: salivary gland; Cell type: glandular cells; Level: None; Expression type: APE; Reliability: Low |
|  | Tissue: seminal vesicle; Cell type: glandular cells; Level: None; Expression type: APE; Reliability: Low |
|  | Tissue: small intestine; Cell type: glandular cells; Level: Low; Expression type: APE; Reliability: Low |
|  | Tissue: smooth muscle; Cell type: smooth muscle cells; Level: None; Expression type: APE; Reliability: Low |
|  | Tissue: spleen; Cell type: cells in red pulp; Level: Medium; Expression type: APE; Reliability: Low |
|  | Tissue: stomach, lower; Cell type: glandular cells; Level: Low; Expression type: APE; Reliability: Low |
|  | Tissue: stomach, upper; Cell type: glandular cells; Level: Low; Expression type: APE; Reliability: Low |
|  | Tissue: testis; Cell type: cells in seminiferus ducts; Level: Low; Expression type: APE; Reliability: Low |
|  | Tissue: testis; Cell type: Leydig cells; Level: None; Expression type: APE; Reliability: Low |
|  | Tissue: thyroid gland; Cell type: glandular cells; Level: Low; Expression type: APE; Reliability: Low |
|  | Tissue: tonsil; Cell type: germinal center cells; Level: Low; Expression type: APE; Reliability: Low |
|  | Tissue: tonsil; Cell type: non-germinal center cells; Level: Low; Expression type: APE; Reliability: Low |
|  | Tissue: uterus, post-menopause; Cell type: cells in endometrial stroma; Level: None; Expression type: APE; Reliability: Low |
|  | Tissue: uterus, post-menopause; Cell type: glandular cells; Level: None; Expression type: APE; Reliability: Low |
|  | Tissue: uterus, pre-menopause; Cell type: cells in endometrial stroma; Level: None; Expression type: APE; Reliability: Low |
|  | Tissue: uterus, pre-menopause; Cell type: glandular cells; Level: Low; Expression type: APE; Reliability: Low |
|  | Tissue: vulva/anal skin; Cell type: epidermal cells; Level: Low; Expression type: APE; Reliability: Low |

---

|  |  |
| --- | --- |
| LocatorString | gscquery:achondroplasia |
|  | pmftq:achondroplasia |

---
